# Infiltrative Basal Cell Carcinoma (iBCC) Adjacent to a Desmoplastic Trichoepithelioma (DTE): A Case Report and Literature Review of iBCCs and DTEs

**DOI:** 10.7759/cureus.61893

**Published:** 2024-06-07

**Authors:** Hannah Khan, Lydiah Mpyisi, Martin J Azzam, Kara Braudis

**Affiliations:** 1 Dermatology, University of Nevada Reno School of Medicine, Reno, USA; 2 Dermatology, University of Missouri School of Medicine, Columbia, USA

**Keywords:** tumor, skin, neoplasm, malignant, infiltrative, desmoplastic trichoepithelioma, carcinoma, basal cell

## Abstract

The co-existence of an infiltrative basal cell carcinoma (iBCC) and a desmoplastic trichoepithelioma (DTE) within the same cutaneous lesion is a rare occurrence. iBCCs are relatively common malignant skin neoplasms that pose a risk for local tissue destruction and recurrence. DTEs are cutaneous neoplasms originating from hair follicles that may clinically and histologically appear similar to iBCCs but are ultimately benign. Distinguishing between these two entities is important given their differing destructive potential. Herein, we describe the case of a 36-year-old female with a single skin lesion on her left cheek that was comprised of both an iBCC distinct from a DTE, as verified by histopathologic analysis. A literature review highlights the rarity of such collision tumors and discusses the potential genetic links between these two histologically similar cutaneous neoplasms.

## Introduction

Basal cell carcinomas (BCCs) are the most common, non-fatal cutaneous malignancies in the United States [1]. If treatment is inadequate or delayed, BCCs can be highly destructive and ultimately disfigure local tissues [1]. There are numerous subtypes of BCCs that appear in the literature, including nodular, superficial, micronodular, and infiltrative, among others. Infiltrative BCCs (iBCCs) have a higher risk for local recurrence than most other subtypes of BCC, which complicates treatment and management. Clinically, iBCCs present as compact or atrophic white/shiny papules and plaques with undefined borders. They are commonly localized to the face or upper trunk [2].

Desmoplastic trichoepitheliomas (DTEs) are relatively uncommon benign cutaneous neoplasms originating from hair follicles that are preferentially localized to the face. DTEs often present as a single discrete white papule with a central indentation on the cheek. Typically, DTEs lack ulceration and are superficial in nature, developing slowly on the face/cheeks of middle-aged females [3].

Below, we present the interesting case of a 36-year-old female with an iBCC adjacent to a DTE on the left nasolabial fold. The initial biopsy of the lesion was equivocal, so further histopathologic analysis was pursued following conservative excision of the entire lesion. It showed angulated nests of basaloid cells with fibrotic stroma in the dermis adjacent to a dermal proliferation composed of cords of small, compact basaloid cells associated with dense fibrocytic stroma. These histologic findings were most consistent with the aforementioned iBCC adjacent to a DTE. The literature has been reviewed for any basis regarding potential links or etiologies between iBCCs and DTEs, as they appear quite similar on histopathologic analysis. This homogeneity likely accounted for the inconclusive nature of the original biopsy specimen.

## Case presentation

A 36-year-old female with no significant past dermatologic history presented to the dermatology clinic for a total-body skin exam. Her only cutaneous concern was a changing bump on her left cheek that occasionally bled when manipulated. The patient reported that it had been present for many years. She indicated a pertinent family history of non-melanoma skin cancer but endorsed good sun protective behavior via wearing a broad-brimmed hat and SPF 30+ sunscreen whenever outdoors for any extended period of time. Past medical history was non-contributory and review of systems was negative.

**Figure 1:**
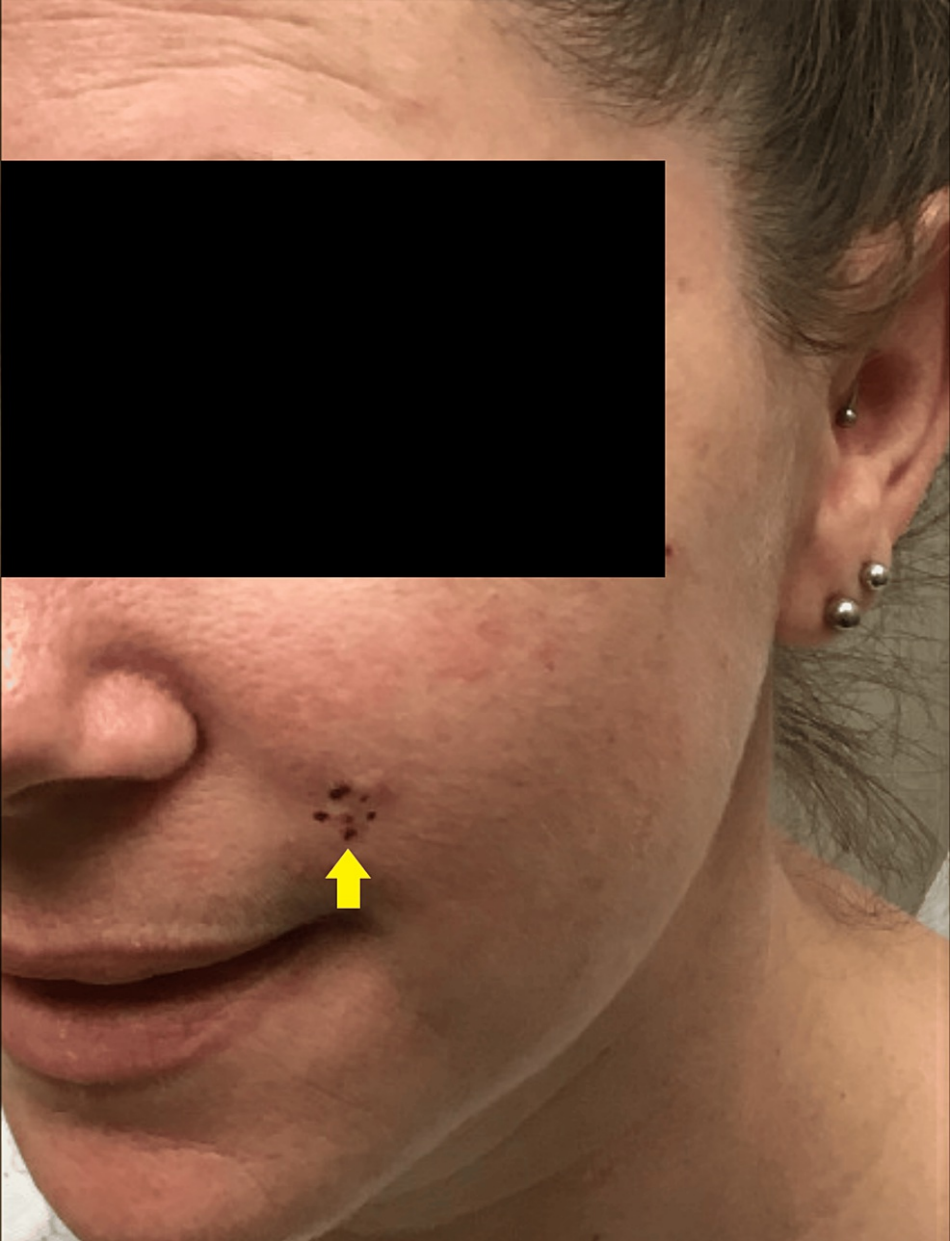
Clinical presentation of a neoplasm of uncertain behavior on the left nasolabial fold of a 36-year-old female prior to the initial biopsy The lesion, outlined in purple ink (yellow arrow), consisted of a 0.6 x 0.4 cm shiny white papule with overlying vessels and visible excoriations on the left cheek at the nasolabial fold.

Physical examination was remarkable for only a 0.6 x 0.4 cm shiny white papule with overlying vessels and visible excoriations on the left cheek at the nasolabial fold (Figure 1). A tangential shave biopsy returned as an unspecified basaloid follicular neoplasm. The dermatopathologist favored DTE; however, complete removal of the lesion was recommended.

**Figure 2:**
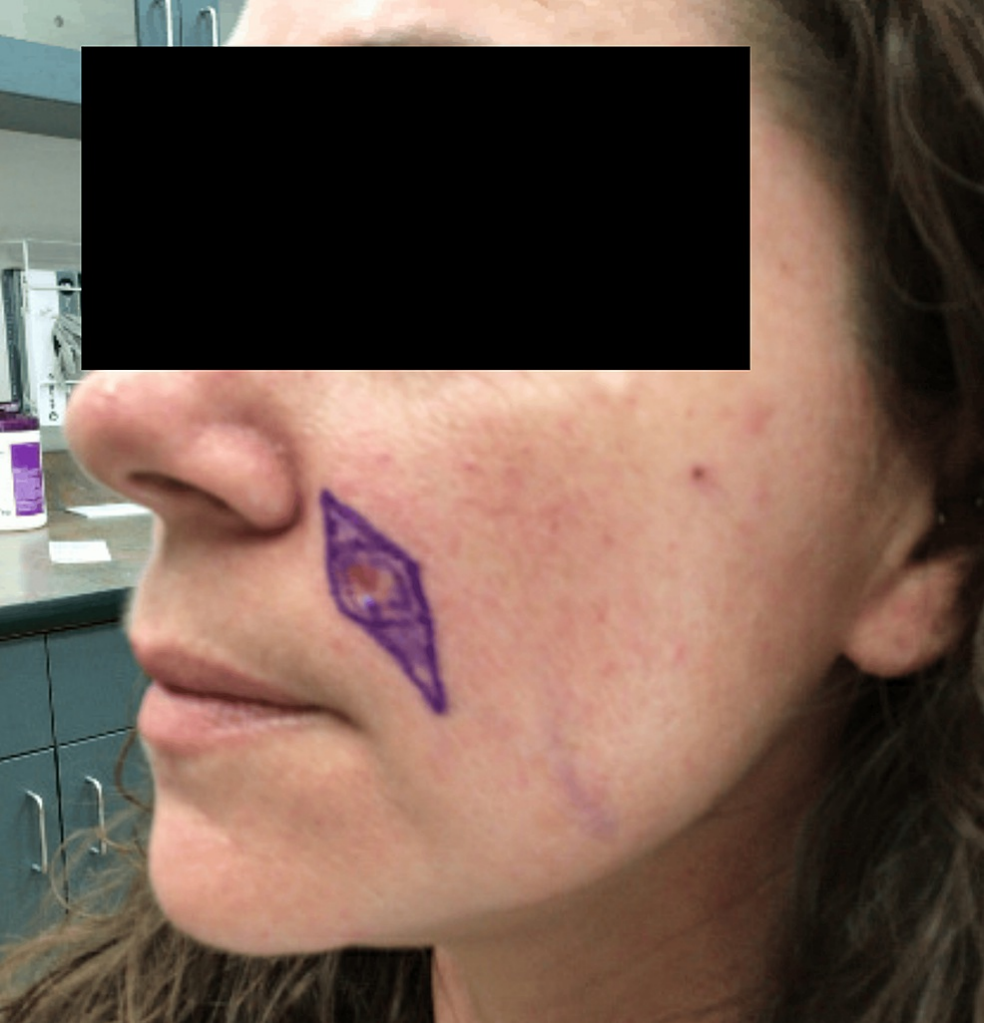
Pre-operative clinical presentation of an unspecified basaloid follicular neoplasm on the left nasolabial fold of a 36-year-old female prior to conservative elliptical excision The lesion, with its conservative excision margins outlined in purple ink, consisted of a 0.8 x 0.7 cm well-healed circular biopsy scar on the left nasolabial fold.

**Figure 3:**
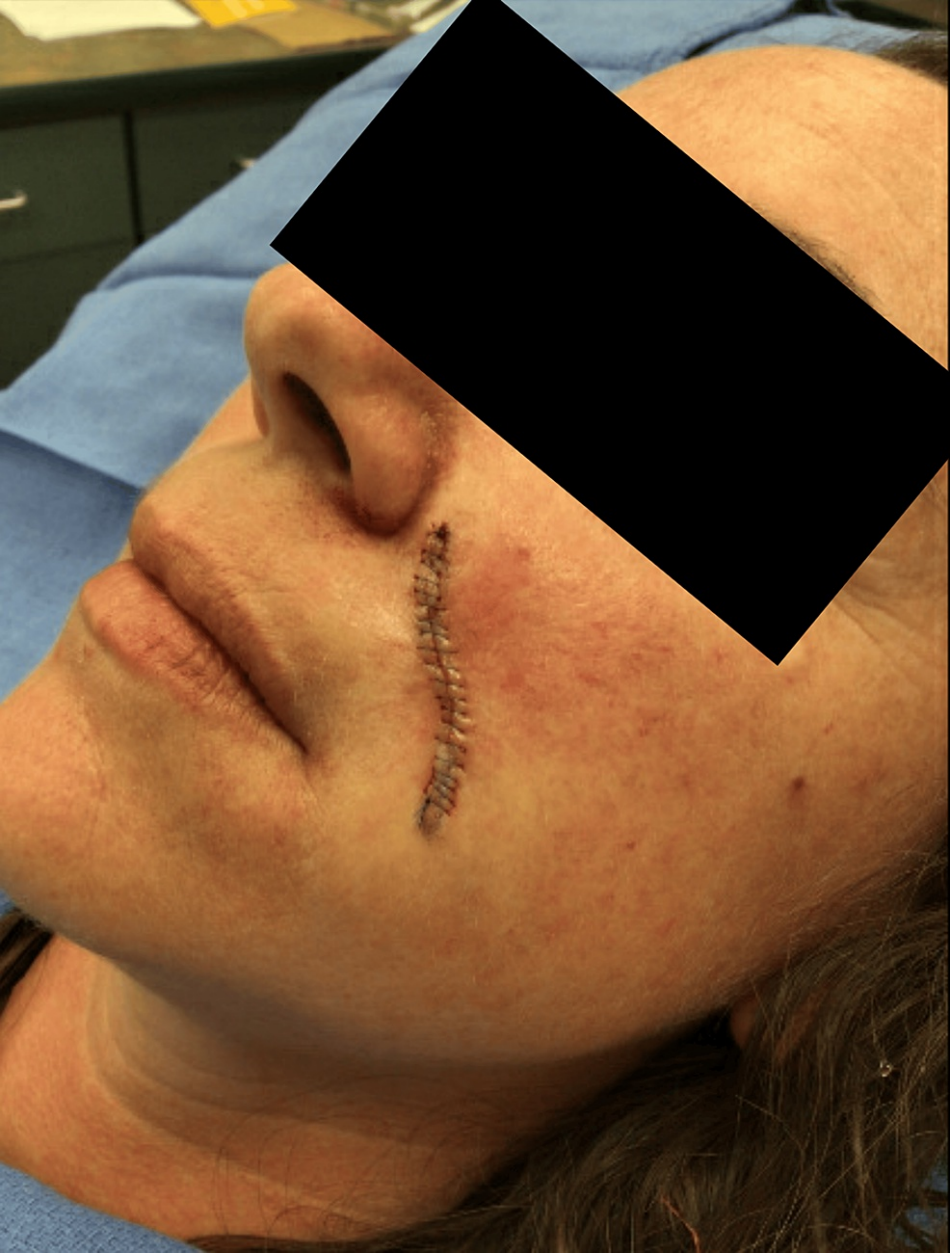
Postoperative appearance of an unspecified basaloid follicular neoplasm on the left nasolabial fold of a 36-year-old female after conservative elliptical excision and intermediate linear layered repair An intermediate linear layered repair with a final size of 3.6 cm was accomplished via the use of 5-0 vicryl deep sutures and 6-0 prolene simple running superficial sutures.

Complete removal of the lesion was undertaken via conservative (e.g., 0.2 cm margins) elliptical excision along the left nasolabial fold, followed by an intermediate linear layered repair (Figures 2, 3).

**Figure 4:**
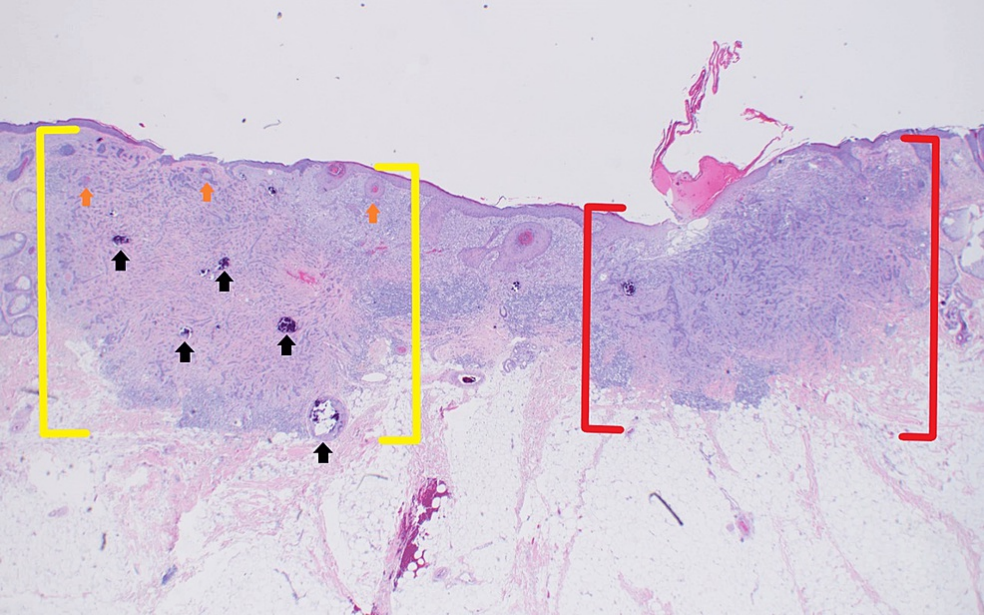
Pathology presentation of an iBCC adjacent to a DTE on the left nasolabial fold of a 36-year-old female; excision specimen Low-power view of an iBCC consisting of angulated nests of basaloid cells with fibrotic stroma (red brackets) adjacent to a DTE (yellow brackets). At this magnification, one can appreciate the small cystic areas of keratinization (orange arrows) and focal calcifications (black arrows) that are characteristic of DTE on the left side of the image above (hematoxylin and eosin: x2). iBCC: infiltrative basal cell carcinoma; DTE: desmoplastic trichoepithelioma

**Figure 5:**
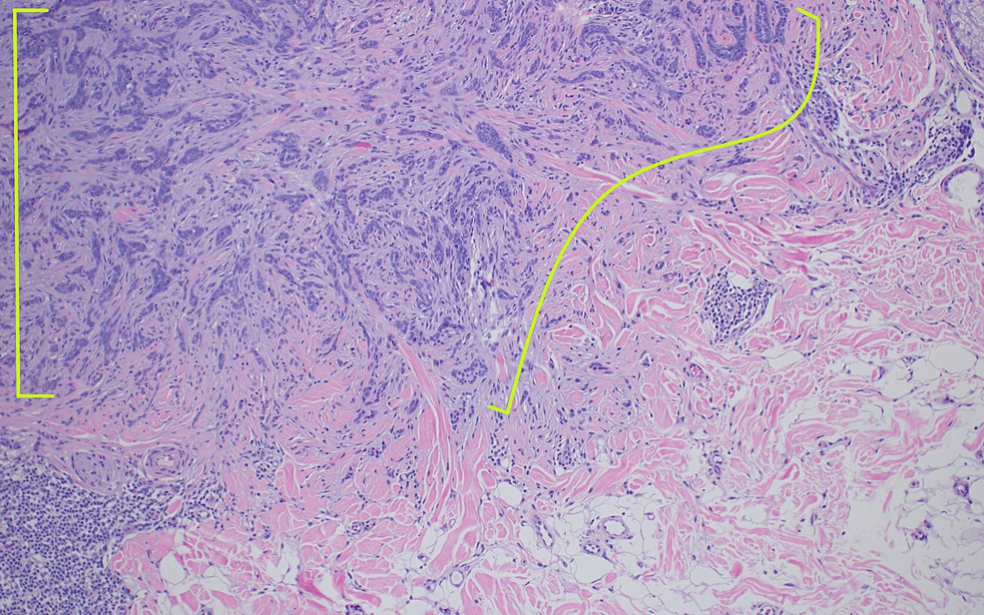
Magnified view of an iBCC within a dermal proliferation on the left nasolabial fold of a 36-year-old female; excision specimen. At this magnification, one can appreciate the nests of angulated blue basaloid cells with scant cytoplasm and elongated hyperchromic nuclei (green brackets) that are characteristic of iBCC on the right side of the dermal proliferation seen in Figure 4 (hematoxylin and eosin: x10). iBCC: infiltrative basal cell carcinoma

Microscopic examination of the completely excised specimen showed a dermal proliferation composed of angulated nests of basaloid cells with fibrotic stroma in the dermis adjacent to a dermal proliferation composed of cords of small, compact basaloid cells associated with dense fibrocytic stroma (Figure 4). Small cystic areas of keratinization and focal calcification were also noted (Figure 4) on one side of the proliferation as were elongated hyperchromic nuclei within angulated nests on the other side (Figure 5).

**Figure 6:**
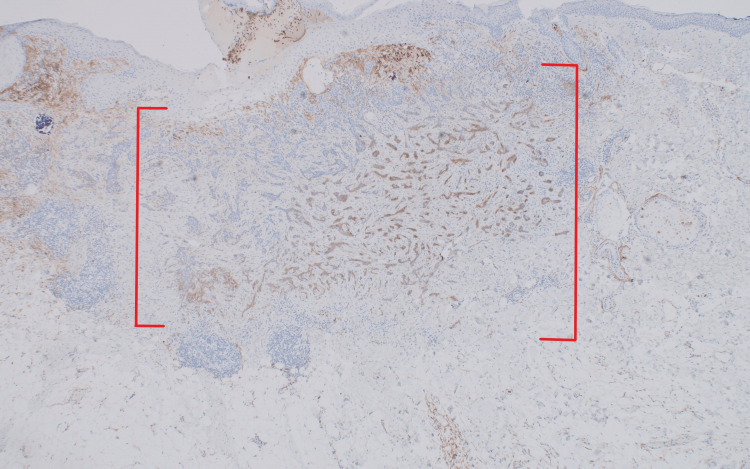
Positive CD10 immunohistochemical confirmation of the iBCC portion of the lesion on the left nasolabial fold of a 36-year-old female There is diffuse CD10 positivity, consistent with iBCC, throughout the angulated nests of basaloid cells within a fibrotic stroma that makes up the iBCC portion of the lesion (red brackets) [x10]. iBCC: infiltrative basal cell carcinoma

**Figure 7:**
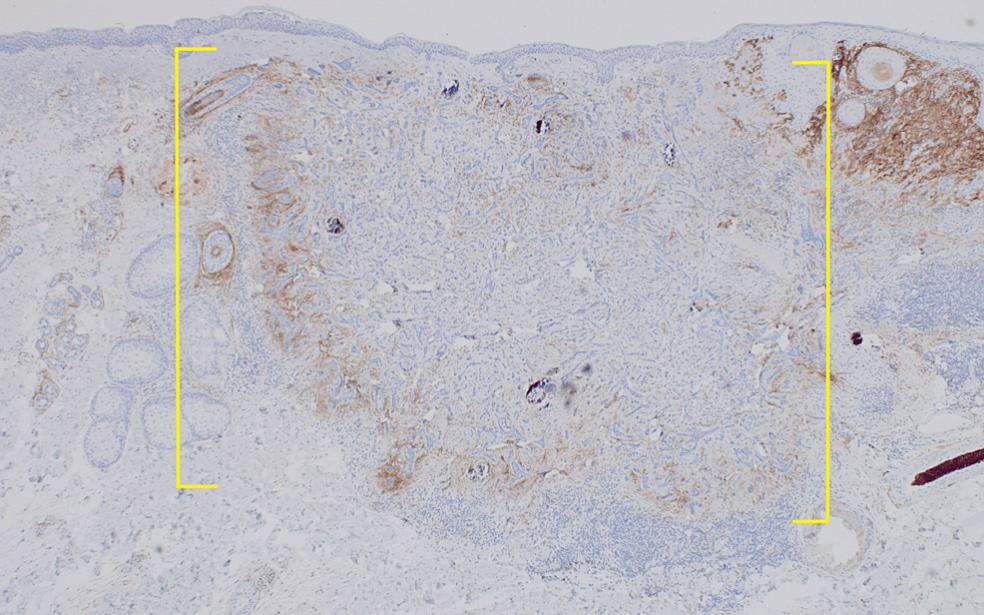
Negative CD10 immunohistochemical confirmation of DTE portion of lesion on the left nasolabial fold of a 36-year-old female There is a lack of CD10 positivity within the cords of small, compact basaloid cells associated with dense fibrocytic stroma that make up the DTE portion of the lesion (yellow brackets) [x10]. iBCC: infiltrative basal cell carcinoma; DTE: desmoplastic trichoepithelioma

Immunohistochemical confirmation of both lesions was achieved with CD10, which is positive in iBCC (Figure 6) and negative in DTE (Figure 7) [4].

At the one-year follow-up, all that remained of the excised iBCC adjacent to a DTE was a well-healed surgical scar hidden within the contours of the patient’s left nasolabial fold. There was no evidence of erythema, pigmentation, induration, nodularity, or recurrence of the lesion.

## Discussion

iBCCs are malignant cutaneous neoplasms that consist of thin angulated bundles of basaloid cells extending into the dermis. They typically present as white/shiny compact or atrophic papules with undefined borders and are commonly localized to the face or upper trunk. Their most common driver mutations include loss-of-function mutations in the gene *PTCH1* (*protein patched homolog 1*) and/or gain-of-function mutations in the gene *SMO *(*smoothened*), both of which are usually induced by ultraviolet radiation [1]. There are many different subtypes of BCC, with the most common being nodular, micronodular, superficial, morpheaform, and infiltrative. The most critical risk factor for all BCCs is chronic ultraviolet light exposure, as it often results in direct and indirect DNA damage, as well as localized immune suppression in the skin. The prognosis of BCCs is dependent upon the risk of recurrence following initial therapy, which can be attributable to location and histopathologic features [2].

DTEs are benign cutaneous adnexal neoplasms that originate from the hair follicles. They typically present as nondescript, firm skin-colored papules with a central depression and are commonly found on the cheeks of younger females. Their most common driver mutations are due to either congenital or acquired loss-of-function mutations in the gene *CYLD *(*CYLD lysine 63 deubiquitinase*) [3]. Additionally, DTEs reveal a lack of CD10 marker (e.g. cluster of differentiation 10 enzyme) expression, a feature that aids in distinguishing them from BCCs for accurate diagnosis, as both these lesions can look similar on histopathology [4].

In our patient’s case, the diagnosis of iBCC and DTE included a thorough history of present illness, detailed clinical background and physical examination, multiple specimens for histopathologic analysis, and appropriate immunohistochemistry to differentiate these histopathologically similar neoplasms from one another. The distinguishing characteristic features of iBCCs and DTEs are described in detail in Table 1.

**Table 1:** Distinguishing characteristic features of iBCC and DTE iBCC: infiltrative basal cell carcinoma; DTE: desmoplastic trichoepithelioma

Distinguishing Characteristics	Infiltrative Basal Cell Carcinoma (iBCC)	Desmoplastic Trichoepithelioma (DTE)
Clinical Features	Pearly or waxy papule or nodule; flesh-colored or brown scar-like lesion on sun-exposed areas often with telangiectasias and rolled borders, or rodent ulcer appearance [5,6].	A slow-growing solitary round papule or nodule, almost exclusively on the face of adult females [3,7]; +/- depression in the center of the lesion; may have elevated borders [8].
Diagnostics	Shave, punch, incisional, or excisional biopsy is recommended [2].	Full-thickness skin biopsy via shave, punch, incisional, or excisional biopsy is advised [9].
Histologic Morphology	Nests of angulated blue basaloid cells with scant cytoplasm and elongated hyperchromic nuclei arising from the basal layer of the epidermis or follicular epithelium with peripheral palisading [1,10].	Tadpole-shaped cords, usually limited to the dermis, however, a connection with the epidermis or hair follicles can also be seen; and could also be associated with keratin-filled cystic spaces and focal calcification [3,10].
Stroma	Stroma displays peritumoral clefting due to retraction artifact, as well as mucin-filled clefts, that separate the pale bluish myxoid stroma from the nests of angulated blue basaloid cells comprising the neoplasm [1,10].	Stroma is densely wrapped around the basaloid cells of this neoplasm; round-to-oval stromal cells push into the basaloid cells, a feature known as papillary mesenchymal bodies (this is a classic finding in younger patients) [3,10].
Stains	Stains diffusely positive for CD10 and focally positive for androgen receptor (AR) [4,10].	Stains negative for CD10; however, scattered benign “passenger” Merkel cells stain positive for CK20 amid the neoplasm’s basaloid cells [4,10].
Treatment	Destruction via curettage, cryotherapy, and/or electrodessication; surgery; radiotherapy [2,10].	Surgery to assure clear margins and non-recurrence [11].
Prognosis	An aggressive subtype of BCC that may lead to substantial local tissue destruction; low (but not 0%) potential for metastasis [10].	True DTEs are benign neoplasms with indolent behavior and minimal malignant potential [3,10].

Furthermore, in our patient’s case, both these histologically similar-appearing neoplasms were discovered within the same clinical lesion. The co-existence of multiple skin neoplasms at the same cutaneous site is a fascinating phenomenon that has been previously referred to as a collision tumor. This occurrence involves two or more benign or malignant neoplasms that are adjacent or intermingled within the same clinical lesion on the skin. The categorization of such tumors is based on the number of neoplasms present at the site, such as dineoplastic (two neoplasms), trineoplastic (three neoplasms), tetraneoplastic (four neoplasms), and so on. This approach to categorization provides a methodical approach based on numerical multipliers defined by the International Union of Pure and Applied Chemistry (IUPAC) [12].

Collision tumors can be further subclassified based on their pathogenetic mechanism(s), which may involve a mixture of different cell clones (clonalium) or the evolution of the same clone of cells (clonalidem). This classification system considers factors such as collision, colonization, combination, and biphenotypic subtypes to understand the interactions between the different neoplasms present within a lesion. In the context of BCC-associated collision tumors, various benign and malignant neoplasms have been reported to co-exist with BCC at the same site. For instance, nevi and seborrheic keratoses are commonly associated with BCC as benign tumors while melanoma in situ and invasive melanoma are frequently observed as malignant tumors alongside BCC [12].

Several documented cases, such as the co-occurrence of syringocystadenoma papilliferum and basal cell carcinoma within a nevus sebaceus on the scalp, highlight the complexity and diagnostic challenges associated with collision tumors [13]. Additionally, a case involving a hidrocystoma and basal cell carcinoma on the eyelid underscores the importance of thorough evaluation, photographic documentation, appropriate biopsy, and histological examination to differentiate between benign and malignant lesions, especially when atypical features are present [14,15].

Lastly, it is worth mentioning that the malignant transformation of trichoepithelioma has previously been documented in the literature, though it is uncommon [3]. One must wonder if there is an as-yet-undiscovered shared mutation driving both neoplastic processes of iBCC and DTE, especially in younger patients. Alternatively, the afflicted cells could have just randomly accumulated these exact mutations spontaneously and ended up in the same collision tumor by chance. Further studies characterizing the genetic profile of each of these neoplasms could yield much useful information regarding the pathogenesis of both common and uncommon neoplasms within collision tumors.

## Conclusions

This case serves to highlight the intriguing co-existence of an infiltrative basal cell carcinoma (iBCC) and a desmoplastic trichoepithelioma (DTE) within the same clinical lesion. This rare occurrence underscores the need for a thorough clinical evaluation, complete histopathology, and an appropriate understanding of the differentiation between various benign and malignant neoplasms, particularly when faced with unique presentations like the one described above. The potential links between iBCCs and DTEs remain a subject of interest as the shared pathway(s) driving these processes, especially in younger patients and collision tumors, merits further investigation. Genetic profiling of these neoplasms offers valuable insights into the underlying pathogenesis of such tumors, paving the way for enhanced diagnostic and therapeutic strategies in the future.
